# Generating Potential Protein-Protein Interaction Inhibitor Molecules Based on Physicochemical Properties

**DOI:** 10.3390/molecules28155652

**Published:** 2023-07-26

**Authors:** Masahito Ohue, Yuki Kojima, Takatsugu Kosugi

**Affiliations:** Department of Computer Science, School of Computing, Tokyo Institute of Technology, Kanagawa 226-8501, Japankosugi@li.c.titech.ac.jp (T.K.)

**Keywords:** protein-protein interaction inhibitor, rule of five, rule of four, QEPPI, molecular generation, virtual chemical library

## Abstract

Protein-protein interactions (PPIs) are associated with various diseases; hence, they are important targets in drug discovery. However, the physicochemical empirical properties of PPI-targeted drugs are distinct from those of conventional small molecule oral pharmaceuticals, which adhere to the ”rule of five (RO5)”. Therefore, developing PPI-targeted drugs using conventional methods, such as molecular generation models, is challenging. In this study, we propose a molecular generation model based on deep reinforcement learning that is specialized for the production of PPI inhibitors. By introducing a scoring function that can represent the properties of PPI inhibitors, we successfully generated potential PPI inhibitor compounds. These newly constructed virtual compounds possess the desired properties for PPI inhibitors, and they show similarity to commercially available PPI libraries. The virtual compounds are freely available as a virtual library.

## 1. Introduction

Significant advances in science and technology have been made since the 1950s; however, the efficiency of the drug discovery process has notably declined. Specifically, the number of drug approvals per billion USD of research and development spending has halved approximately every nine years [1]. It has also been reported that the cost of bringing a drug to market can now be up to 2.8 billion USD [2]. One of the main reasons for this decline in drug discovery is that the current drug-target space is nearly saturated. Therefore, since the early 2000s, researchers have been actively exploring novel therapeutic targets, such as protein-protein interactions (PPIs) [3,4,5,6,7]. PPIs play a crucial role in various cellular processes and are associated with many diseases, such as cancer [7] and Alzheimer’s disease [8]. However, targeting PPIs is complex, and only a few compounds have been approved or have progressed to the clinical trial stage as PPI inhibitors [9].

One of the difficulties in targeting PPIs is that they have different properties compared to conventional drug discovery targets. The binding interface of PPIs is wider than the average binding region of a typical protein target. As a result, PPI inhibitor molecules tend to be larger and more diverse in conformation [10].

Lipinski et al. [11,12] proposed a rule of thumb, known as the “Rule of Five (RO5)”, to evaluate the likelihood of a compound with specific physicochemical properties becoming an orally active drug. According to this rule, the compounds that do not meet two or more of the following four criteria are considered to be poorly absorbed and unlikely to eventually become pharmaceutical products.

- Molecular weight ≤ 500
- Indicator of lipophilicity, LogP ≤ 5
- Number of hydrogen bond donors ≤ 10
- Number of hydrogen bond acceptors ≤ 5.

On the other hand, Morelli et al. [13] studied the properties of PPI inhibitors obtained from the 2P2I database [14]. This database contains protein complex structures and interaction regions stored in the PDB file format, along with complex structures where PPI inhibitors are bound. They showed that most PPI inhibitors from the database violated RO5 significantly. Furthermore, they showed that the following rule of thumb, called the “Rule of Four (RO4)”, was more applicable to the PPI inhibitors.

- Molecular weight > 400
- Indicator of lipophilicity, LogP > 4
- Number of cyclic structures > 4
- Number of hydrogen bond acceptors > 4

High throughput screening (HTS) is commonly used to find active compounds in the early phases of drug discovery. HTS is a rapid assay process that evaluates numerous compounds quickly and can effectively identify active compounds from vast libraries for a specific target. However, when HTS is performed for PPI as a target using a compound library consisting of general small molecule compounds, the acquisition rate of hit compounds is significantly lower [15]. Therefore, studies on the development of PPI-specific libraries to obtain high hit rates have been conducted [16,17]. However, such existing compound libraries mainly contain derivatives of the principal core structures used in known PPI inhibitors, limiting the diversity of compounds in the libraries.

To address this restricted range of compounds, we focused on a large-scale search method in this study. Moreover, we used a deep-reinforcement learning-based molecular generation model to produce potential novel PPI inhibitors. Molecular generation aims at discovering novel compounds with desirable properties and activities from a vast compound space. To produce potential PPI inhibitors, the scoring function of the molecular generation model was modified according to their properties. In addition, a virtual library containing the generated PPI-target compounds was constructed.

## 2. Results

### 2.1. Inducing Exploration through Reinforcement Learning

**Figure 1:**
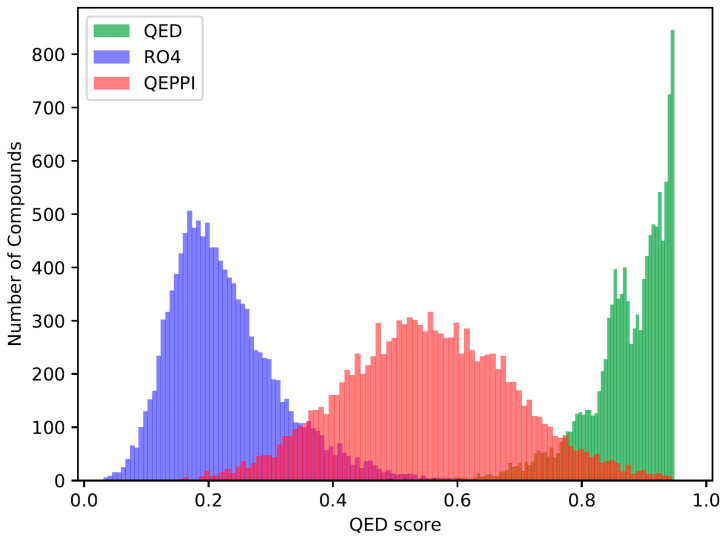
Distribution of QED scores of the compounds generated between the REINVENT steps 2901 and 3000.

**Figure 2:**
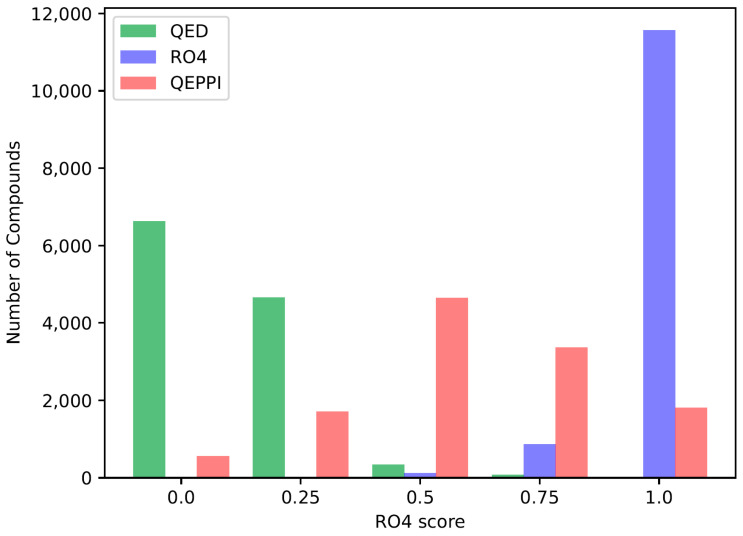
Distribution of RO4 equivalent scores of the compounds generated between the REINVENT steps 2901 and 3000.

**Figure 3:**
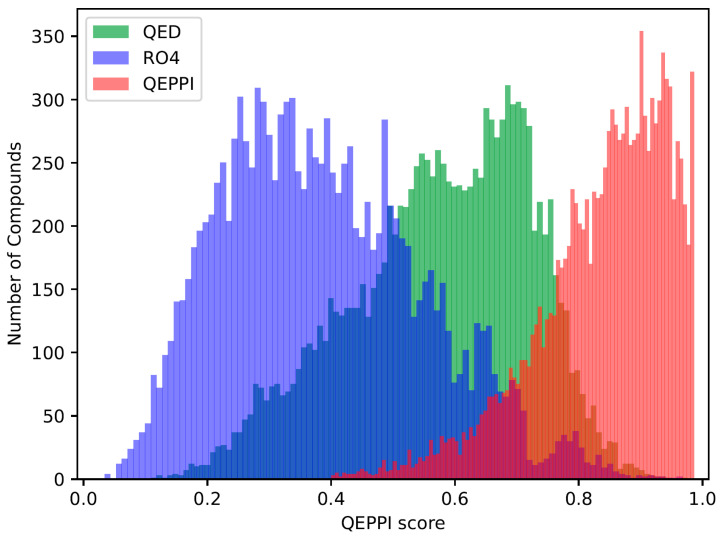
Distribution of QEPPI scores of the compounds generated between the REINVENT steps 2901 and 3000.

The quantitative estimates of drug likeness (QED) [18], RO4 equivalent, and the quantitative estimate index for early-stage screening of compounds targeting PPIs (QEPPI) [19] scores were calculated for each of the three generated compound sets, and their distributions for each score are shown in Figure 1, Figure 2 and Figure 3. As anticipated and in line with expectations, the investigation confirmed that compounds generated with QED as the scoring function tend to have higher QED scores (Figure 1), compounds generated with RO4 as the scoring function tend to have higher RO4 scores (Figure 2), and compounds generated with QEPPI as the scoring function tend to have higher QEPPI scores (Figure 3). As can be seen, the exploration was induced to the region where the scoring function was higher, indicating that reinforcement learning was properly performed.

### 2.2. Distribution of Compounds Generated by REINVENT

**Figure 4:**
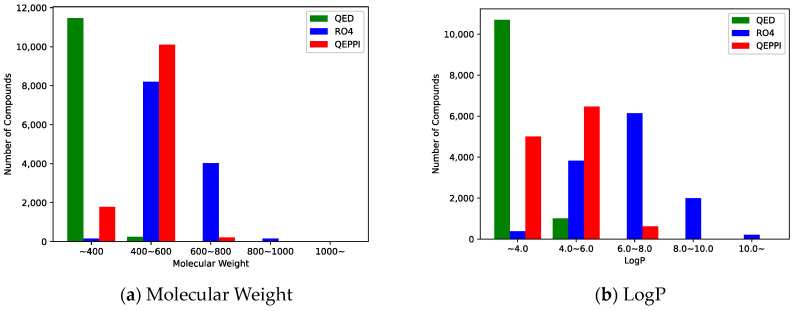
Distribution of the generated compounds: (**a**) distribution of molecular weight, (**b**) distribution of lipophilicity (LogP).

**Figure 5:**
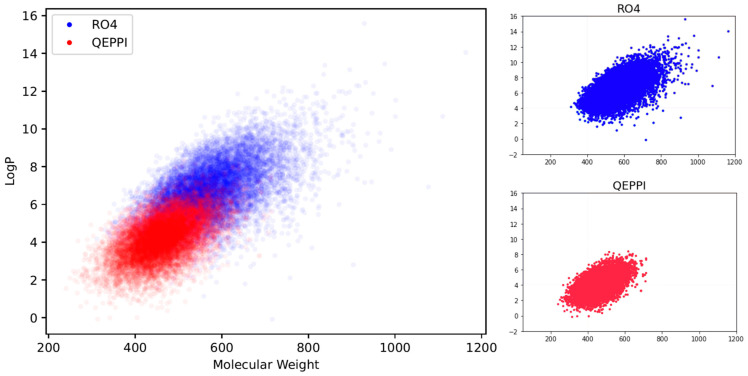
Molecular weight–LogP scatter plots of the RO4- and QEPPI-generated compounds.

Molecular weight and LogP, a measure of lipophilicity, are both related to RO4 and can be used to evaluate PPI inhibitor likeness. Figure 4a,b shows the distribution of molecular weight and LogP of the generated compounds for each scoring function. In the case of QED, both molecular weight and LogP were skewed toward the smaller values, and the generated molecules could not be suitable for PPI inhibitors. On the other hand, when RO4 was used, most of the molecules generated satisfied RO4 (molecular weight > 400, LogP > 4). Although the QEPPI-generated molecules did not satisfy RO4, 85.3% and 58.6% of all the generated compounds fulfilled RO4 conditions for molecular weight and LogP, respectively. In addition, Figure 5 plots a two-dimensional scatterplot of the overlaid molecular weight and LogP distributions for the RO4 and QEPPI cases. Molecules with excessively high molecular weight and LogP, such as in RO4, were not generated by the QEPPI scoring function, and the compounds were concentrated in the appropriate chemical space where high QEPPI scores can be obtained.

### 2.3. Indicators for Oral Bioavailability

Veber’s rule is an indicator of oral bioavailability, and compounds that satisfy the following two properties tend to have good membrane permeability when administered orally [20].

- Number of rotatable bonds, Rbond ≤ 10
- Topological polar surface area TPSA ≤ 140

The numbers of QED-, RO4-, and QEPPI-generated molecules satisfying Veber’s rule were 11,712, 9617, and 12,029, with percentages of 99.9% (11,712/11,714), 76.6% (9617/12,547), and 99.4% (12,029/12,097), respectively. Thus, molecules generated using the QEPPI scoring function can also be expected to have good oral bioavailability at ratios almost equal to those generated using the QED.

### 2.4. Constructing Virtual Libraries of PPI-Target Compounds

Based on the results, the compounds generated based on the QEPPI are more likely to be able to target PPIs, as they combine general drug-like properties while satisfying PPI inhibitor-specific properties, such as RO4. Therefore, 12,097 compounds generated based on QEPPI were used as the basis for the virtual library for developing PPI inhibitors. 

**Figure 6:**
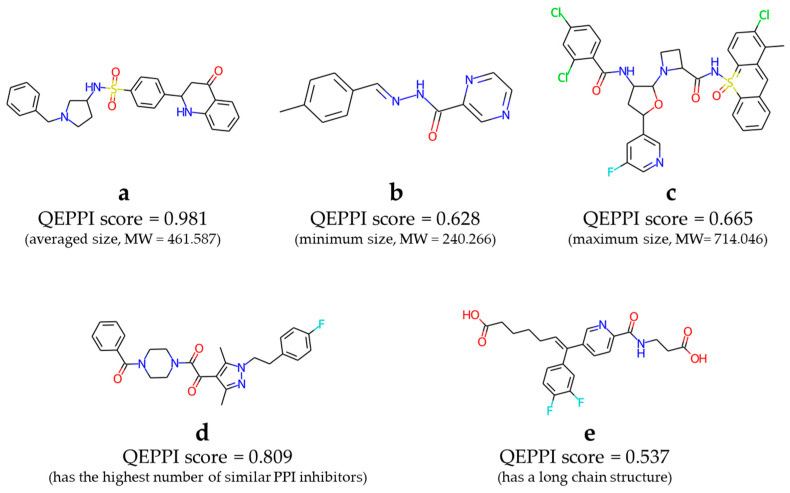
Examples of compounds that have been generated and included in the virtual library.

In addition, the pan assay interference compounds (PAINS) filter [21] was used to identify substructural features of generated compounds that appear in promiscuous compounds or frequent hitters in many biochemical high-throughput screening campaigns and removed their compounds. The PAINS filter removed 845 (7.0%) compounds, and then the remaining 11,252 compounds constituted the virtual library, published on https://github.com/ohuelab/iPPI-REINVENT (accessed on 5 July 2023). Figure 6 shows some examples of the compounds included in this library (Supplementary Figures S1–S3 show more examples). Compounds **a**, **b**, and **c** in Figure 6 had the average, minimum, and maximum molecular weights, respectively, of the compounds in the virtual library. Compound **d** had the highest number of similar known PPI inhibitors. Compound **e** is an example of a compound containing a slightly longer alkyl chain. In fact, these compounds will not be suitable as drugs as they are, especially **c**, which has a strange structure with an aromatic ring containing a sulfonamide. However, we hope that PPI inhibitor hits will be obtained from this virtual library and contribute to drug discovery.

## 3. Discussion

### 3.1. Chemical Space of Generated Compounds

One of the objectives of this study was to generate novel PPI inhibitor compound candidates from an unexplored compound space by modifying a scoring function according to the characteristics of PPI inhibitors in the molecule generation model. In this study, three types of scoring functions, QED, RO4, and QEPPI, were used to explore the compound space and generate molecules. The distributions of molecular weight and LogP for each compound shown in Figure 4 and Figure 5 are skewed toward the smaller values for QED and are excessively large for RO4. On the other hand, the QEPPI-generated molecules exhibited smaller and more coherent distributions while satisfying RO4. Therefore, the QEPPI-based molecular generation achieved the above objective by exploring the compound space that remained unexplored in the cases of QED and RO4. Note that the pre-trained model used in this study utilized ChEMBL and was not PPI inhibitor specific. Therefore, pre-training with a dataset derived from a known PPI inhibitor may improve the efficiency of the exploration and generation.

### 3.2. Comparison with Known PPI Modulators

To ascertain that compounds generated by QEPPI possess properties more suitable for PPI inhibition, we conducted a search for PPI modulators with a Tanimoto similarity > 0.5 to the compounds generated by QED, RO4, and QEPPI, as characterized by ECFP4 fingerprints. The search targeted 2426 manually curated non-peptide PPI modulators from iPPI-DB [22]. As a result, 25 similar PPI modulators were found from 11,714 compounds generated by QED, 29 from 12,547 compounds generated by RO4, and 120 from 12,097 compounds generated by QEPPI. QEPPI identified more than four times the number of similar PPI modulators compared to the scoring by QED or RO4. These findings suggest that the compounds produced by QEPPI display a tendency more similar to existing PPI modulators compared to those generated by QED or RO4, implying divergent structural trends.

### 3.3. Comparison with Existing PPI Libraries

To determine whether the QEPPI-based compound library, which is considered more suitable for recent PPI inhibitor design, could be a useful virtual library, we compared the corresponding 12,097 compounds with those in an existing PPI compound library—the Enamine PPI library [23]. This commercial PPI library contains 40,640 compounds with core structures that are expected to bind to specific substructures extracted from more than 20 different protein complex structures. Supplementary Figure S4 shows some example compounds of this library.

**Figure 7:**
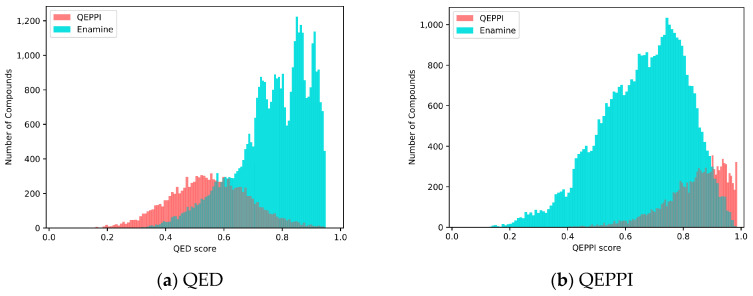
Distribution of scores (number of steps: 100) for the compounds generated by the QEPPI and obtained from the Enamine PPI library: (**a**) QED and (**b**) QEPPI scores.

**Figure 8:**
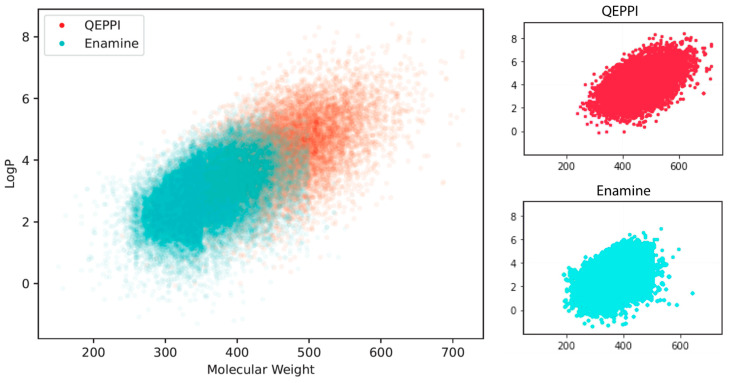
Molecular weight–LogP scatterplots of the compounds generated by the QEPPI and obtained from the Enamine PPI library.

Figure 7a,b shows the distributions of the QED and QEPPI scores. In addition, Figure 8 shows two-dimensional scatterplots for molecular weight and LogP. These results show that the compounds in the Enamine library have small molecular weights and LogP values and high QED scores, while those generated by QEPPI have higher molecular weights and LogP values. Moreover, the QEPPI scores of the compounds generated in this study were also higher. Therefore, we can conclude that commercial PPI libraries are oriented toward more oral drug-like tendencies and that our proposed generated compounds contain more PPI inhibitor-like compounds.

### 3.4. Limitations and Future Directions

The synthesizability of virtual compounds is also crucial for actual biochemical assays. The PAINS filter was applied to the generated compounds to remove inappropriate compounds; however, this method alone cannot consider synthetic feasibility. In fact, in the Enamine PPI library, those compounds that fit all of the multiple medicinal chemistry filters, including PAINS, were selected [23]; for example, the synthetic accessibility score [24] guesses the difficulty of synthesizing a compound. There is also room for consideration of a more reliable retrosynthetic analysis [25], although it requires long computation times. In future work, we would like to incorporate such a method that allows us to computationally evaluate the synthetic feasibility of compounds. It would be necessary to consider and provide the contribution of molecular substructures with interpretable artificial intelligence and other technologies [26].

Additionally, while the compounds generated by QEPPI tend to have high bioavailability, the generation of PPI inhibiting compounds with even greater potential as oral drugs remains a crucial challenge. As QED and QEPPI share similar constituent descriptors, adjusting the weights of each descriptor used in QEPPI could alter the composition of the generated compounds. This could potentially enable the generation of compounds that possess both oral bioavailability and PPI inhibition properties.

Finally, techniques that enhance inhibitory capabilities against actual targets are also important. Although this study does not target specific target proteins or target PPIs, there exist scoring schemes [27] that target specific targets and compound generation methods [28,29] that include protein-ligand docking simulations. By combining with structure-based drug discovery, it is expected that the efficiency of drug development can be improved [30]. We aim to construct an even more helpful chemical library that will enable efficient screening in the future.

## 4. Materials and Methods

In this study, we used a molecular generation model called REINVENT (version 3.0), developed by Blaschke et al. [31]. This model is based on character string (SMILES notation) and uses recurrent neural networks as the architecture. Moreover, it generates molecules with desired properties in combination with reinforcement learning. A pre-trained model based on ChEMBL [32], which is a database of chemical compounds, was obtained from [33] and used for reinforcement learning in the same way as described in a previous study [31].

The scoring function of REINVENT *S*(*x*) of one generated compound *x* is calculated in the range of 0 to 1. Three scoring functions used in this study were QED [18], RO4 [13] and QEPPI [19]. QED is an index that quantifies the drug likeness of small molecules, and is equivalent to making RO5 continuous. It is defined in the range of 0 to 1, and it was used as the score *S*(*x*). RO4 was introduced as the REINVENT score, and for each of the fulfilled RO4 four conditions, 0.25 is added to the total score *S*(*x*); thus, the score ranges from 0 to 1. The QEPPI is a quantitative index of PPI inhibitor suitability, ranging from 0 to 1. It has also been used for PPI inhibitor evaluation in molecular generation studies [34]. The QEPPI was used directly as the overall score *S*(*x*) in this study.

The three scoring functions, QED-based, RO4-based, and QEPPI-based, described above, were used in this work, and reinforcement learning was performed for 3000 steps each. Parameters other than the scoring function *S*(*x*) were set to default values [33]. The numbers of compounds generated by applying the QED, RO4, and QEPPI scoring functions in 3000 steps of REINVENT training were 357,456, 368,140, and 359,722, respectively. To obtain higher scoring molecules among the generated compounds, the compounds generated in the last 100 steps (i.e., between steps 2901 and 3000) were extracted, resulting in 11,714, 12,547, and 12,097 compounds for QED, RO4, and QEPPI, respectively.

## 5. Conclusions

In this study, we used REINVENT—a machine-learning-based molecular generation tool—to generate compounds using three types of scoring functions: QED, RO4, and QEPPI. In all cases, we could generate compounds with the desired properties targeted by the scoring functions. In the context of this study, which aimed at generating candidate compounds for novel PPI inhibitors, compounds generated by QED- and RO4-based function did not prove suitable. However, compounds generated by QEPPI-based function demonstrated more fitting properties. After filtering out compounds with inappropriate structures from this QEPPI-based generated compounds using the PAINS filter, a total of 11,252 compounds were made available as a PPI virtual library. The expectation is for the discovery of new PPI inhibitor lead compounds among these virtual compounds.

Only the QEPPI-generated compounds were considered in this study. Since REINVENT supports complex scoring functions, we believe that the search for compounds in chemical space can be broadened by applying and modifying various scoring functions. In future work, we intend to explore ways to enhance the scoring functions and expand our search to include chemical spaces that cannot be adequately covered by the existing PPI libraries.

## Data Availability

Data on the compounds generated and codes created in this study are provided at https://github.com/ohuelab/iPPI-REINVENT (accessed on 5 July 2023).

## Supplementary Materials

The following supporting information can be downloaded at: https://www.mdpi.com/article/10.3390/molecules28155652/s1, Figure S1: QED-based generated molecules; Figure S2: RO4-based generated molecules; Figure S3: QEPPI-based generated molecules; Figure S4: Compounds from Enamine PPI library.
