# Supplementary material for: Generating Potential Protein-Protein Interaction Inhibitor Molecules Based on Physicochemical Properties
Source: Molecules. 2023 Jul 26;28(15):5652. doi: 10.3390/molecules28155652 (PMC10420264; doi:10.3390/molecules28155652)
Supplement: Supplementary file 1 [file molecules-28-05652-s001.zip › molecules-2490133-supplementary.pdf]

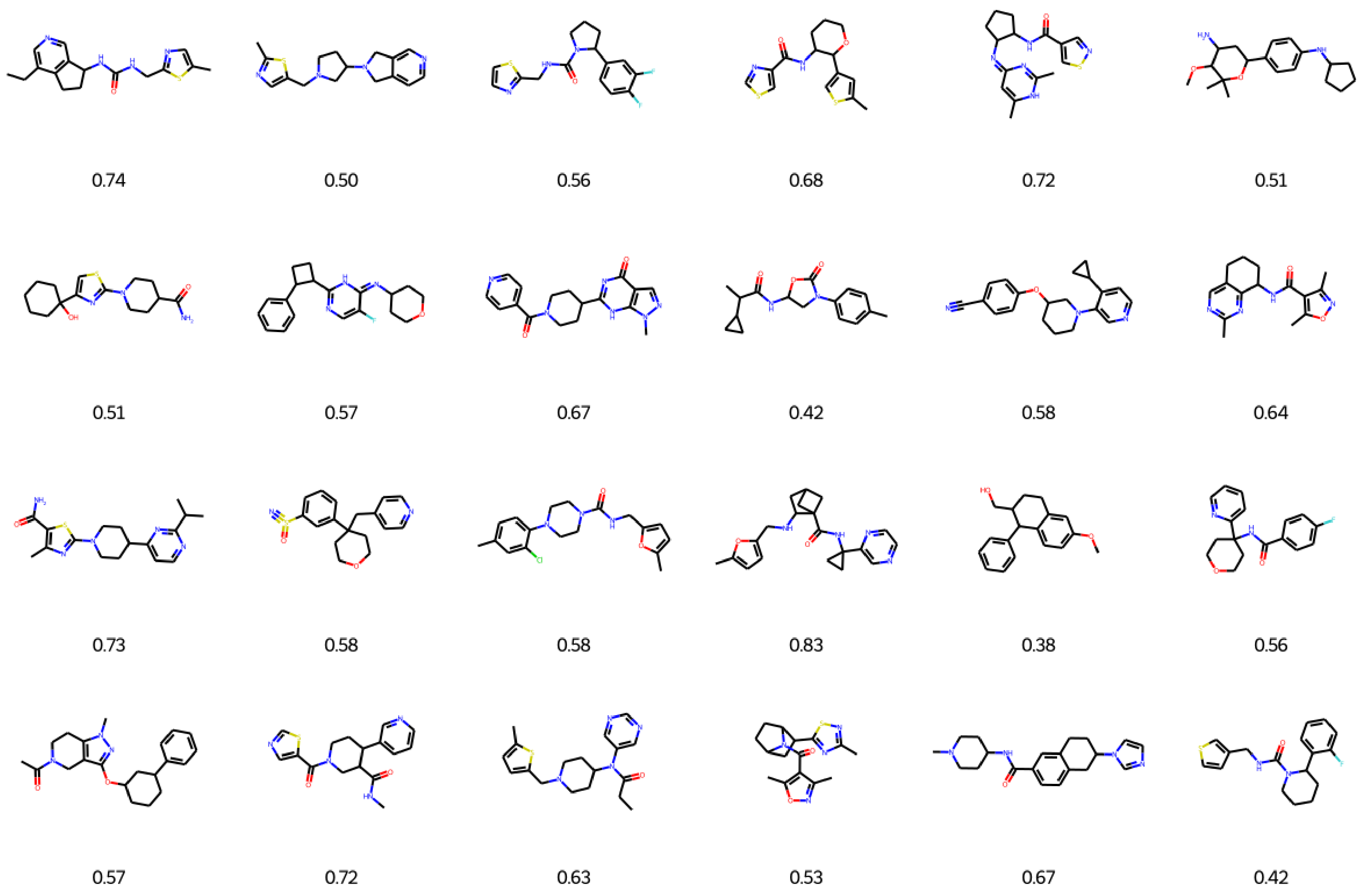

Supplementary Figure S1: **QED-based generated molecules.**
These compounds were randomly extracted from the library created. The value under the compound is the QEPPI score.

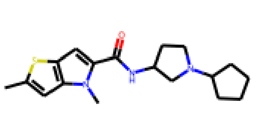

0.62

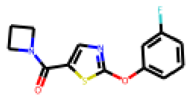

0.51

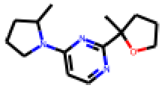

0.31

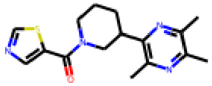

0.55

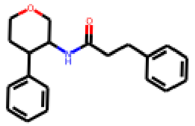

0.46

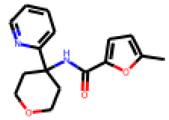

0.67

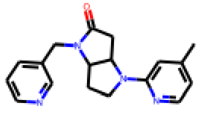

0.54

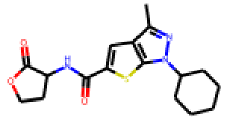

0.70

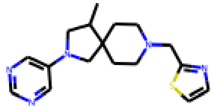

0.53

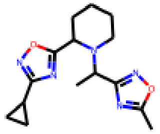

0.53

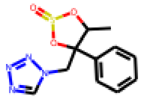

0.47

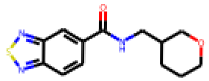

0.68

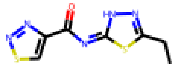

0.56

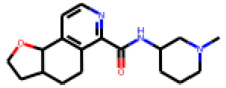

0.43

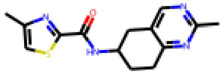

0.64

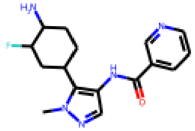

0.72

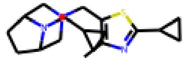

0.34

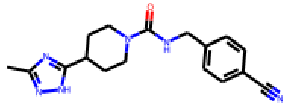

0.69

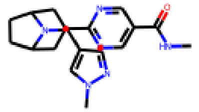

0.72

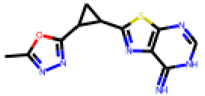

0.56

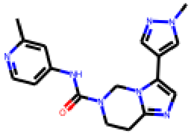

0.70

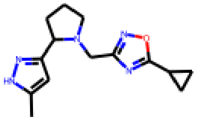

0.72

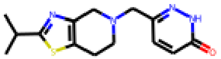

0.68

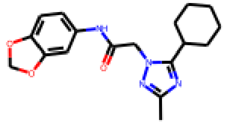

0.73

Supplementary Figure S1 (cont’d)

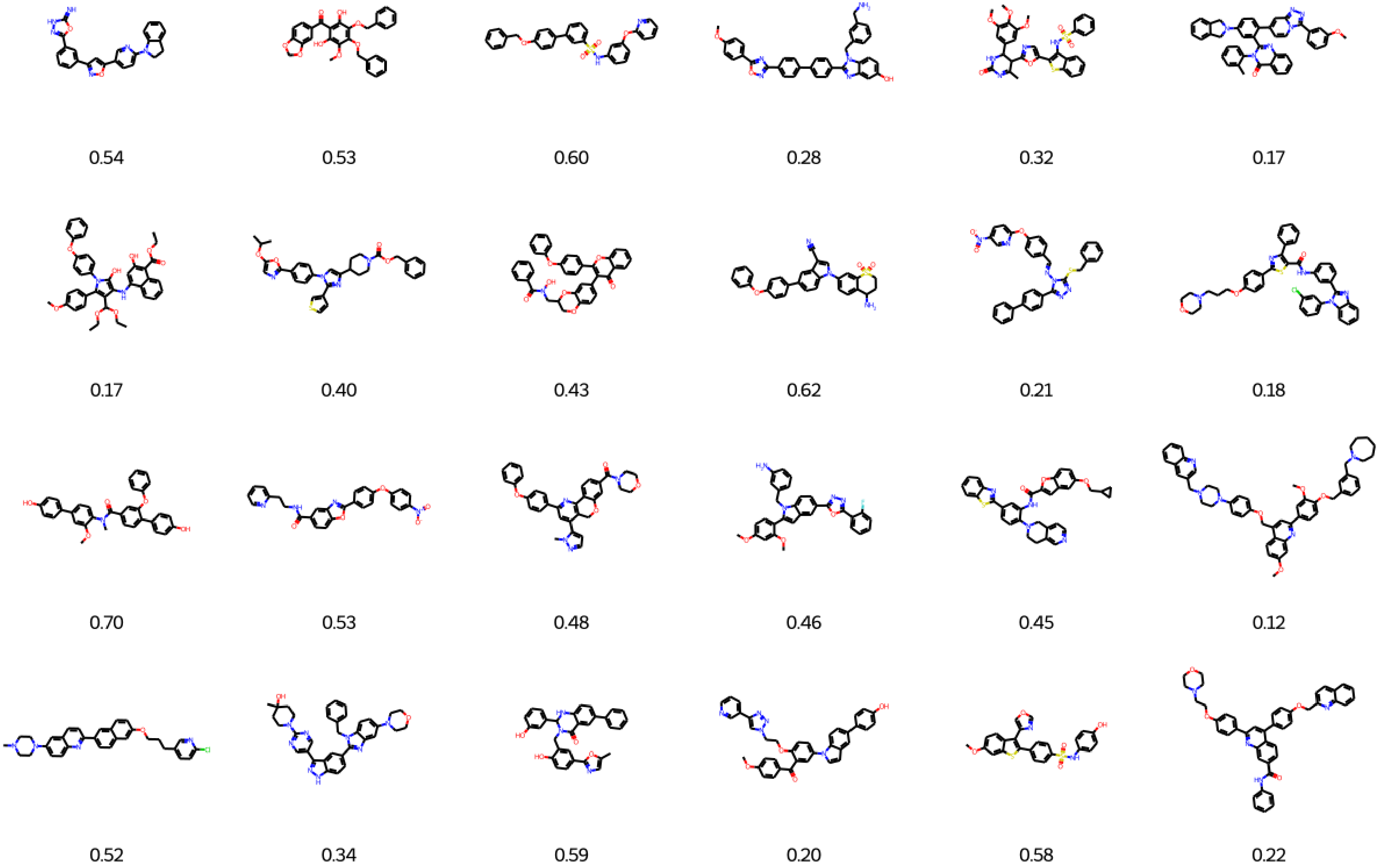

Supplementary Figure S2: **RO4-based generated molecules.**
These compounds were randomly extracted from the library created. The value under the compound is the QEPPI score.

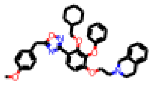

0.28

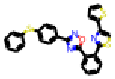

0.36

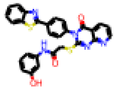

0.35

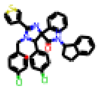

0.55

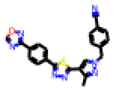

0.36

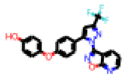

0.56

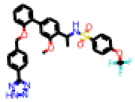

0.33

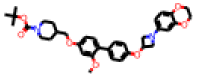

0.54

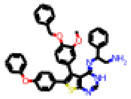

0.23

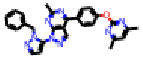

0.30

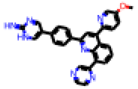

0.45

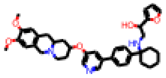

0.45

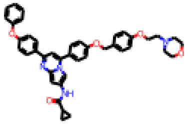

0.26

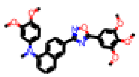

0.32

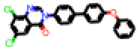

0.50

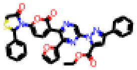

0.20

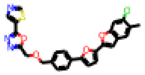

0.34

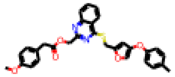

0.33

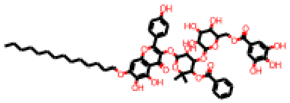

0.08

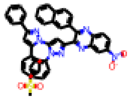

0.31

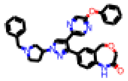

0.50

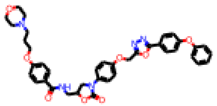

0.21

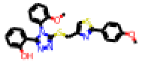

0.44

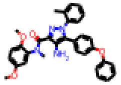

0.55

Supplementary Figure S2 (cont'd)

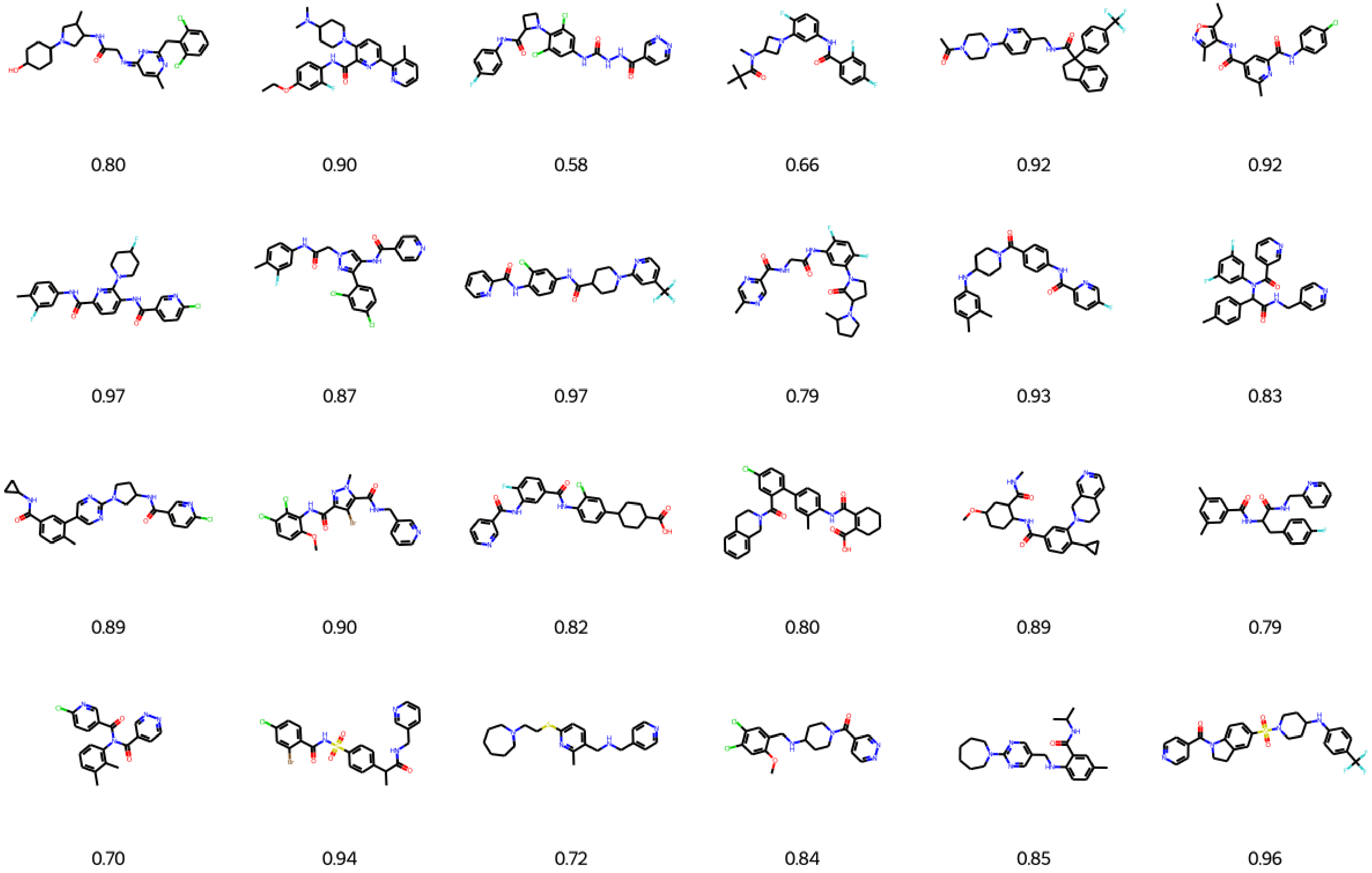

Supplementary Figure S3: **QEPPI-based generated molecules.**
These compounds were randomly extracted from the library created. The value under the compound is the QEPPI score.

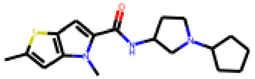

0.62

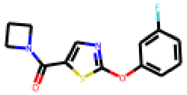

0.51

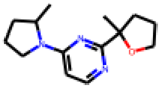

0.31

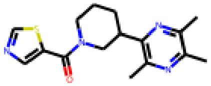

0.55

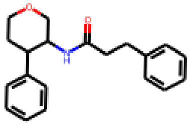

0.46

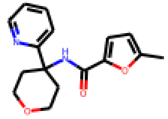

0.67

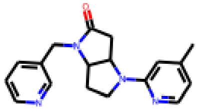

0.54

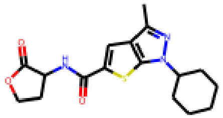

0.70

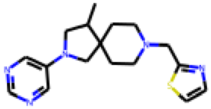

0.53

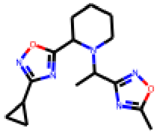

0.53

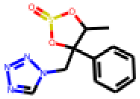

0.47

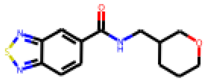

0.68

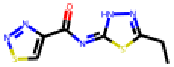

0.56

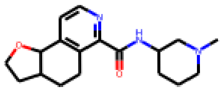

0.43

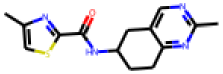

0.64

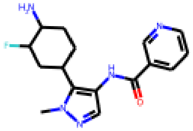

0.72

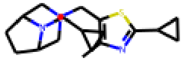

0.34

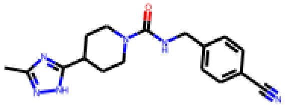

0.69

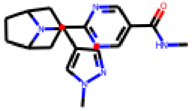

0.72

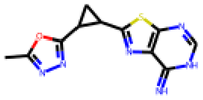

0.56

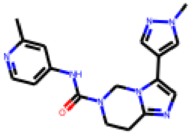

0.70

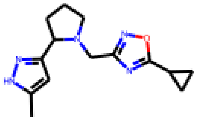

0.72

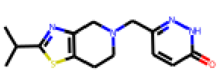

0.68

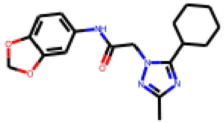

0.73

Supplementary Figure S3 (cont'd)

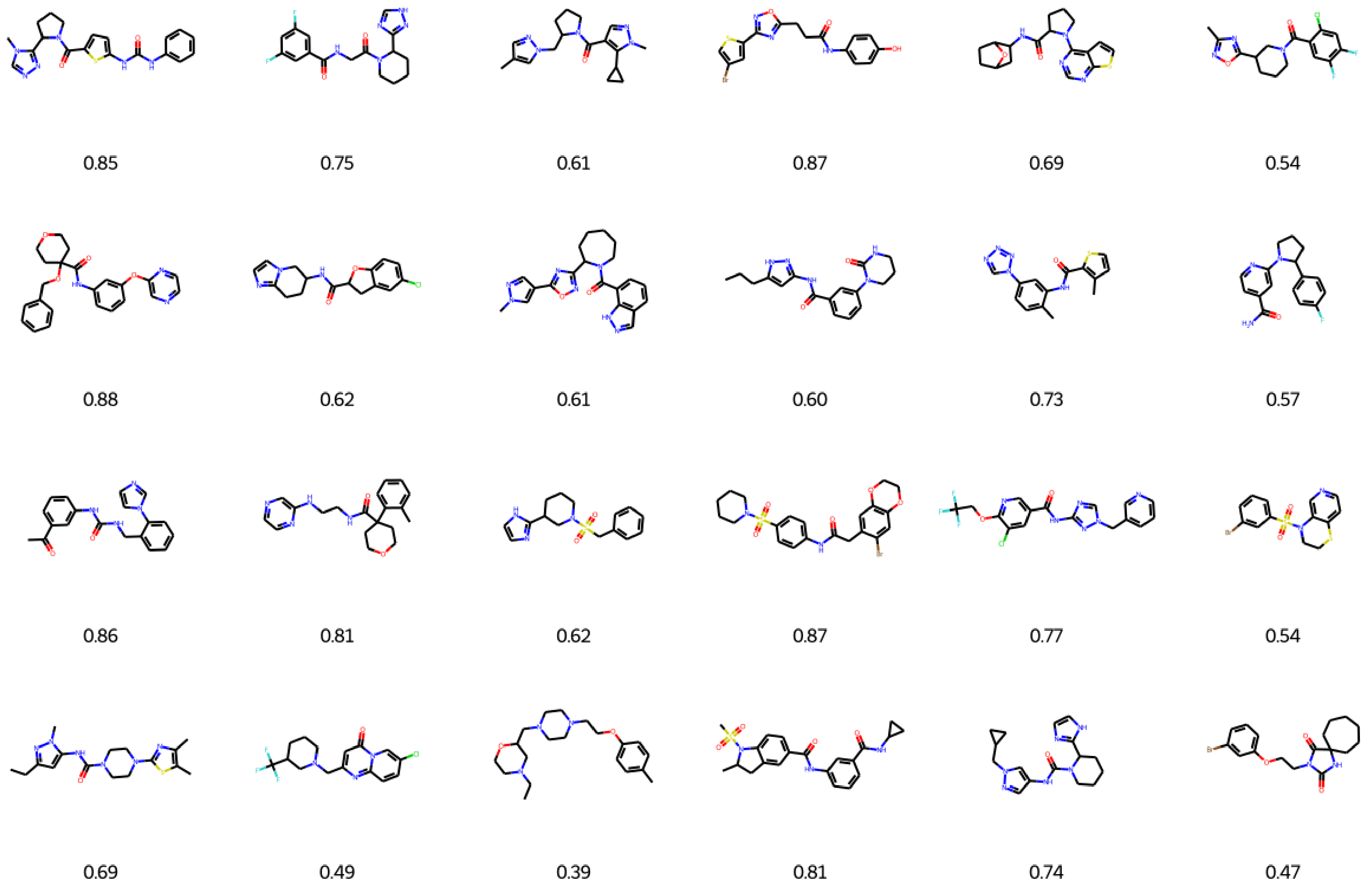

Supplementary Figure S4: **Compounds from Enamine PPI library.**
These compounds were randomly extracted from the library. The value under the compound is the QEPPI score.

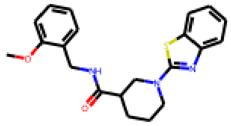

0.88

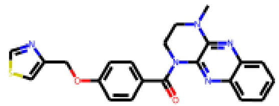

0.57

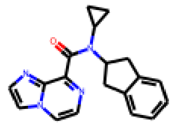

0.61

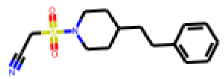

0.37

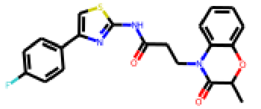

0.94

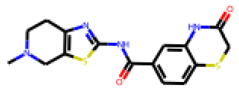

0.66

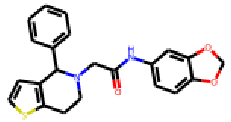

0.85

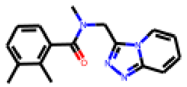

0.59

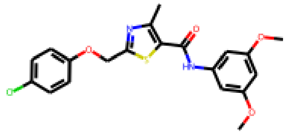

0.89

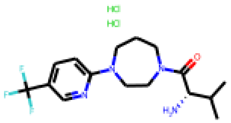

0.53

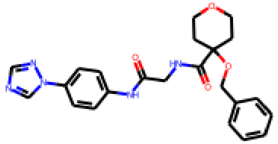

0.74

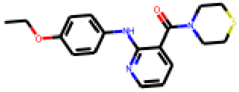

0.77

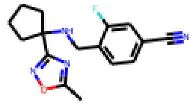

0.75

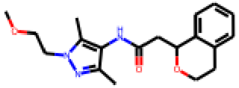

0.80

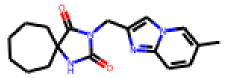

0.65

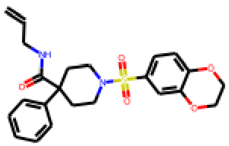

0.86

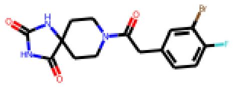

0.42

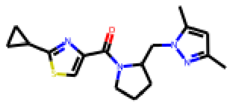

0.61

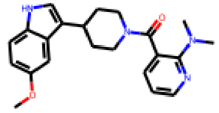

0.85

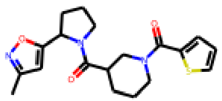

0.64

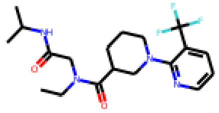

0.57

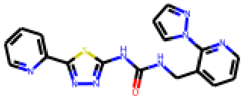

0.57

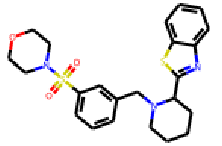

0.74

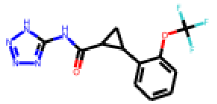

0.75

Supplementary Figure S4 (cont'd)
